# Hydranencephaly in Neonate With Prenatal Exposure to Alcohol During Pregnancy

**DOI:** 10.1002/ccr3.72783

**Published:** 2026-05-24

**Authors:** Dibya Raj Chaudhary, Diwakar Koirala, Samiksha Lamichhane, Bivek Mishra

**Affiliations:** ^1^ BP Koirala Institute of Health Sciences Dharan Nepal

**Keywords:** central nervous system (CNS), choroid plexus coagulation (CPC), congenital, hydranencephaly, hydrocephalus (HCP), ventriculoperitoneal shunt (VP shunt)

## Abstract

Hydranencephaly (HE) is a rare congenital condition characterized by near‐total absence of the cerebral hemispheres, replaced by cerebrospinal fluid, most commonly resulting from in utero bilateral internal carotid artery occlusion; although maternal alcohol consumption is associated with fetal neurodevelopmental abnormalities, its direct role in HE remains unclear. We report a case of a 42‐year‐old multiparous woman with chronic alcohol use throughout pregnancy who delivered a female neonate at 40 + 5 weeks via spontaneous vaginal delivery. The neonate presented with facial asymmetry and a boggy parietal swelling, while maintaining intact reflexes and normal muscle tone. Third‐trimester ultrasonography had suggested hydrocephalus, and postnatal cranial ultrasonography confirmed extensive cerebral destruction with fluid replacing most brain structures. Laboratory investigations, including TORCH screening, were unremarkable. The infant was managed with supportive care and parental counseling. Hydranencephaly is typically associated with early childhood mortality, although rare cases of prolonged survival have been described. Neuroimaging is essential for differentiating HE from severe hydrocephalus and porencephaly, and management remains largely supportive, with ventriculoperitoneal shunting considered in selected cases. This case highlights the importance of early prenatal detection, multidisciplinary management, and increased awareness of potential alcohol‐related fetal brain abnormalities to guide counseling and optimize outcomes.

## Key Clinical Message

Hydranencephaly, a rare congenital disorder, involves absent cerebral hemispheres replaced by fluid, often due to prenatal alcohol exposure. This case of a neonate born to a mother with chronic alcohol use highlights the need for early prenatal diagnosis, imaging, and multidisciplinary care to manage outcomes and support families.

**Abbreviations**
CNS: Central nervous system
CPC: Choroid plexus coagulation
CT: Computed tomography
EBV: Epstein–barr virus
HCP: Hydrocephalus
HE: Hydranencephaly
HSV: Herpes simplex virus
ICAs: Internal carotid arteries
LFT: Liver function test
MRI: Magnetic resonance imaging
NICU: Neonatal intensive care unit
NMDA: N‐methyl‐D‐aspartate (receptor)
RFT: Renal function test
RSV: Respiratory syncytial virus
SVD: Spontaneous vaginal delivery
USG: Ultrasonography
VP Shunt: Ventriculoperitoneal shunt

## 1 Introduction

Hydranencephaly (HE) is a rare congenital disorder marked by the absence of the cerebral hemispheres, which are replaced by a large, cerebrospinal fluid‐filled cavity [1]. It is a severe congenital anomaly primarily resulting from bilateral occlusion of the internal carotid arteries in utero, most commonly occurring during the second trimester of gestation. Its underlying causes encompass infectious, toxic, genetic, and vascular factors, along with maternal complications such as hypoxia or twin‐to‐twin transfusion syndrome. Diagnosis is primarily based on advanced imaging modalities, particularly fetal ultrasound, which can identify a large fluid‐filled cavity devoid of cortical structures, with confirmation typically achieved through MRI or CT postnatally [2]. It is an exceptionally rare and distinctive congenital abnormality, with an estimated prevalence of fewer than 1 in 10,000 births globally [2]. This neurological malformation frequently leads to fetal demise, making postnatal occurrences uncommon [3]. Alcohol consumption can lead to long‐term neurological alterations, including brain atrophy, and may contribute to both acute and chronic neurological disorders, some of which can be life‐threatening. Alcohol‐induced brain shrinkage is linked to the loss of neurons in specific regions, a process mediated by the alteration of N‐methyl‐D‐aspartate (NMDA) receptors [4]. Managing this condition may necessitate surgical procedures, including the placement of a ventriculoperitoneal shunt or a combination of choroid plexus coagulation (CPC) and ventriculostomy, to address complications related to intracranial hypertension [5].

In this case report, we present a case of hydranencephaly secondary to chronic maternal alcohol abuse.

## 2 Case History and Examination

A 42‐year‐old multiparous woman (gravida 4, para 4) delivered a female infant via spontaneous vaginal delivery (SVD) at 40 weeks and 5 days of gestation. The newborn had a birth weight of 3010 g and a head circumference of 34 cm (median to +1Z score). The pregnancy was non‐consanguineous and regularly monitored, with third‐trimester ultrasonography (USG) revealing fetal hydrocephalus with hypertension complicating pregnancy without features of imminent eclampsia in the mother. The pregnancy was reportedly supervised at a local health center with routine antenatal visits. First‐trimester and second‐trimester ultrasonography were not documented and formal anomaly scan reports were unavailable at the time of admission. Fetal movements were perceived normally throughout pregnancy. There was no history of maternal fever, rash, or exposure to teratogenic medications apart from chronic alcohol intake. There was no history suggestive of gestational diabetes or preeclampsia.

At birth, the neonate exhibited a boggy swelling in the parietal region, deviation of the mouth to the right side, and an absent nasolabial fold on the left side. However, the infant was active, with normal muscle tone and intact newborn reflexes. The neonate had an APGAR score of 7 and 9 at 1 and 5 min, respectively. There was no respiratory distress, and oxygen saturation was maintained on room air. Neurological examination revealed preserved primitive reflexes including Moro, rooting, and grasp reflexes. No seizures were observed during hospitalization. Neonatal genetic testing was not done due to resource limitations. However, no dysmorphic features suggestive of a syndromic etiology were observed clinically.

A detailed prenatal history revealed that the mother had consumed approximately 300 mL of locally brewed homemade alcohol, known as “jaad” (containing approximately 20% alcohol), both before and throughout the pregnancy.

## 3 Differential Diagnosis, Investigation, and Treatment

### 3.1 D/D

1. Severe Hydrocephalus: Excessive CSF enlarges ventricles, thinning cortex; distinguishable by preserved cortical rim unlike hydranencephaly's near‐total cerebral absence.
2. Porencephaly: Localized brain cysts from prenatal injury; less extensive than hydranencephaly, with variable neurological impact.
3. Schizencephaly: Gray matter‐lined clefts in hemispheres; substantial brain tissue remains, unlike hydranencephaly's CSF replacement.
4. Holoprosencephaly: Incomplete forebrain division; single‐lobed brain with midline defects, contrasting hydranencephaly's destructive etiology.
5. Congenital CMV Infection: Neuronal loss with calcifications; some brain persists, differing from hydranencephaly's extensive cortical absence.

### 3.2 Investigations and Treatment

**FIGURE 1:**
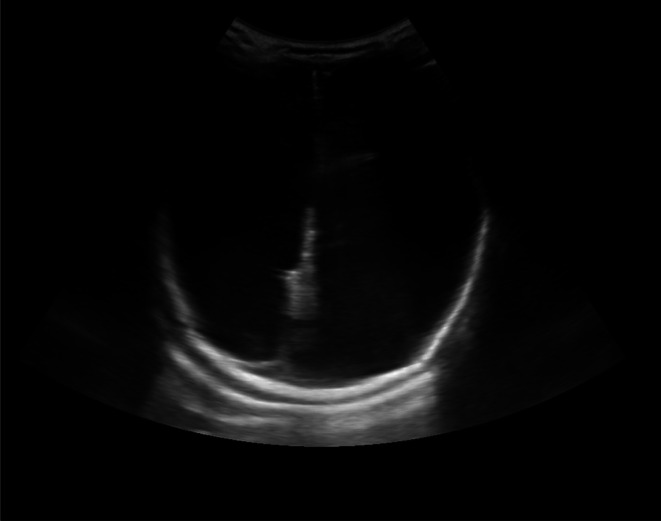
Ultrasound cranium axial view showing dilated ventricle falx is visualized.

**FIGURE 2:**
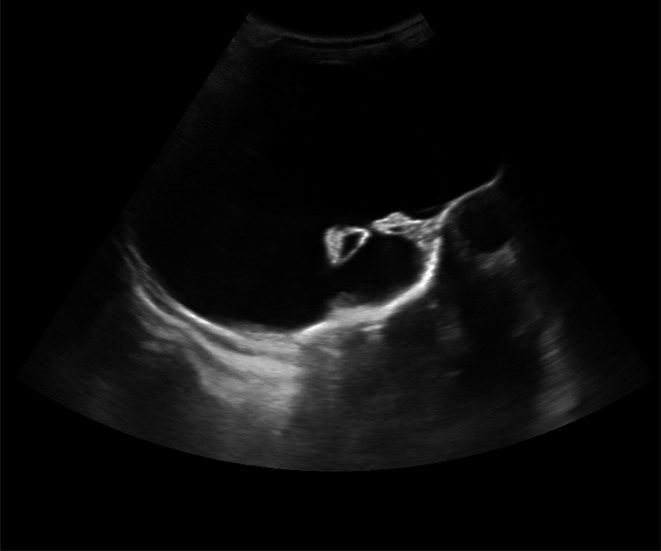
Cranial ultrasound sagittal view with non‐visualization of bilateral cerebral hemisphere which is replaced by fluid.

The infant was admitted to the neonatal nursery for further evaluation of facial asymmetry. Blood investigations, including a complete blood count, liver function tests (LFT), and renal function tests (RFT), were within normal limits. Furthermore, tests conducted for intrauterine viral infections, including TORCH screening, yielded normal results. Cranial ultrasonography demonstrated near‐complete replacement of the brain parenchyma by fluid, with significantly dilated ventricles. The frontal, parietal, temporal, and occipital brain regions could not be visualized, as illustrated in Figures 1 and 2. Additionally, echocardiographic examination revealed no abnormalities.

Ventriculoperitoneal shunting was offered as a treatment modality, but the patient's guardian declined due to financial reasons and was counseled regarding the prognosis of the case.

### 3.3 Conclusion and Results

Hydranencephaly is a rare congenital malformation with highly variable clinical presentations, depending on the severity of the condition. Prenatal ultrasound is typically sufficient for diagnosis, while MRI or intrauterine CT can provide additional insights but are not considered first‐line diagnostic tools. Despite advances in imaging, management remains complex due to ethical and clinical challenges, particularly regarding therapeutic termination. A comprehensive understanding of its etiopathogenesis, along with a multidisciplinary approach, is crucial for optimizing care and supporting affected families.

Early diagnosis plays a vital role in preventing obstetric complications and ensuring appropriate parental counseling during pregnancy. It also facilitates the preparation of optimal delivery conditions, including access to a specialized pediatric care unit at birth. Given the severity of hydranencephaly, timely intervention is essential for improving clinical outcomes and providing the best possible support to both the child and their family.

## 4 Discussion

Hydranencephaly is an encephaloclastic disorder characterized by the absence of the cerebral hemispheres, which are replaced by cerebrospinal fluid and necrotic debris, enclosed by the leptomeninges. In most cases, the cerebral cortex is absent; however, partial preservation of the occipital lobe may occur. The midbrain, thalamus, basal ganglia, choroid plexus, cerebellum, and brainstem are typically intact and remain within the skull. The falx cerebri is usually present but may be partially or completely absent, and the septum pellucidum may also be absent [2].

The etiopathogenesis of hydranencephaly is diverse, with multiple theories proposed to explain its occurrence. The most widely recognized cause is the occlusion of the supraclinoid segment of the bilateral internal carotid arteries, leading to ischemic degeneration of the structures they supply.

Myers [6] investigated the etiology of hydranencephaly through experimental studies on laboratory monkeys. In his research, fetal monkeys underwent ligation of the bilateral carotid arteries and jugular veins at varying gestational stages. The fetuses were then returned to the uterus, allowed to reach full term, and subsequently delivered. Postnatal examination of their brains demonstrated hydranencephaly, with the condition being more pronounced when vascular obstruction occurred at earlier stages of gestation.

Maternal exposure to carbon monoxide or butane gas has been identified as a potential cause of hydranencephaly. Such exposure can lead to fetal hypoxia, which results in widespread tissue necrosis, cavitation, resorption of necrotic tissue, and necrotizing vasculitis [7].

Other etiologies of hydranencephaly include intrauterine infections, which can lead to localized brain tissue destruction. Examples of such infections include congenital toxoplasmosis [8] and various viral agents, such as adenovirus, cytomegalovirus, enterovirus, Epstein–Barr virus, herpes simplex virus [9], parvovirus, and respiratory syncytial virus [10].

In addition, some case reports suggest that a temporary spasm, rather than direct occlusion, of the internal carotid arteries may lead to ischemic damage [11] in certain brain structures, contributing to the development of hydranencephaly.

Hydranencephaly should be distinguished from conditions such as severe hydrocephalus, alobar holoprosencephaly, and porencephaly in the differential diagnosis. On ultrasonography, it typically presents as a large cystic structure devoid of cerebral cortex, which helps differentiate it from other neurological abnormalities [12]. Additionally, hydranencephaly may be associated with various anomalies, including cerebellar abnormalities, ocular malformations, intracranial calcifications, renal dysplasia, and genetic syndromes such as Fowler syndrome.

Ventriculoperitoneal shunting is a commonly employed intervention for managing hydranencephaly by controlling head circumference; however, it carries a risk of complications, including infections. As an alternative, choroid plexus cauterization is sometimes utilized to mitigate shunt‐related complications [13].

Aydin et al. [3] described a case of hydranencephaly in a 22‐year‐old female working in the auto industry with negative TORCH infection and no history of alcohol intake. The 3.3‐kg infant was managed with antiseizure medications, VP shunting, and intubation following transfer to PICU.

Similarly, Toumi [5] et al. described a case of hydranencephaly in a twin pregnancy with the other twin atrophied and non‐viable. The living twin presented with hydranencephaly and hydrocephalus and was scheduled for management with psychological support to the family and regular follow‐up for emerging complications. The different cases have been compared in Table 1.

**TABLE 1:** Comparison of different cases.

Author	Maternal risk factor	Gestational age	Imaging modality	Management
Aydin et al. [3]	Toluene exposure	Term	USG and CT	VP shunt
Toumi et al. [5]	Twin pregnancy	Preterm	Prenatal USG	Supportive
Khalid et al. [1]	Not identified	6 months age	CT	VP shunt
Present case	Chronic alcohol	40 + 5 weeks	Post‐natal USG	Supportive (couldn't afford VP shunt)

Hydranencephaly is most frequently diagnosed during the second half of pregnancy; however, there have been reports of sonographic detection as early as the first trimester. The condition carries a poor prognosis due to the extensive loss of brainstem function. While some affected neonates may not survive at birth, the majority succumb within the first year of life. In rare cases where survival extends beyond infancy, severe neurological impairment is inevitable [2].

## Author Contributions

**Dibya Raj Chaudhary:** conceptualization, data curation, formal analysis, funding acquisition, validation, visualization, writing – original draft, writing – review and editing. **Diwakar Koirala:** methodology, resources, software, validation, visualization, writing – original draft, writing – review and editing. **Samiksha Lamichhane:** visualization, writing – original draft, writing – review and editing. **Bivek Mishra:** visualization, writing – original draft, writing – review and editing.

## Funding

The authors have nothing to report.

## Ethics Statement

Ethical approval was taken for the submission of case report.

## Consent

Written informed consent was obtained from the patient's parents for publication and any accompanying images. A copy of the written consent is available for review by the Editor‐in‐Chief of this journal on request.

## Conflicts of Interest

The authors declare no conflicts of interest.

## Data Availability

Data sharing not applicable to this article as no datasets were generated or analysed during the current study.
